# Discovery of a highly potent glucocorticoid for asthma treatment

**DOI:** 10.1038/celldisc.2015.35

**Published:** 2015-12-15

**Authors:** Yuanzheng He, Jingjing Shi, Wei Yi, Xin Ren, Xiang Gao, Jianshuang Li, Nanyan Wu, Kevin Weaver, Qian Xie, Sok Kean Khoo, Tao Yang, Xiaozhu Huang, Karsten Melcher, H Eric Xu

**Affiliations:** 1 Laboratory of Structural Sciences, Van Andel Research Institute, 333 Bostwick Avenue Northeast, Grand Rapids, MI, USA; 2 VARI-SIMM Center, Center for Structure and Function of Drug Targets, Key Laboratory of Receptor Research, Shanghai Institute of Materia Medica, Chinese Academy of Sciences, Shanghai, China; 3 Department of Medicine, Lung Biology Center, University of California, San Francisco, San Francisco, CA, USA; 4 Laboratory of Skeletal Biology, Van Andel Research Institute, Grand Rapids, MI, USA; 5 College of Life Sciences, Wuhan University, Wuhan, Hubei, China; 6 Molecular Oncogenesis and Targeted Therapy, Laboratory of Molecular Oncology, Center of Cancer and Cell Biology, Van Andel Research Institute, Grand Rapids, MI, USA; 7 Department of Cell & Molecular Biology, Grand Valley State University, Grand Rapids, MI, USA

**Keywords:** Glucocorticoids, glucocorticoid receptor, VSGC12, potency, asthma

## Abstract

Glucocorticoids are the most effective treatment for asthma. However, their clinical applications are limited by low efficacy in severe asthma and by undesired side effects associated with high dose or prolonged use. The most successful approach to overcome these limitations has been the development of highly potent glucocorticoids that can be delivered to the lungs by inhalation to achieve local efficacy with minimal systemic effects. On the basis of our previous structural studies, we designed and developed a highly potent glucocorticoid, VSGC12, which showed an improved anti-inflammation activity in both cell-based reporter assays and cytokine inhibition experiments, as well as in a gene expression profiling of mouse macrophage RAW264.7 cells. In a mouse asthma model, VSGC12 delivered a higher efficacy than fluticasone furoate, a leading clinical compound, in many categories including histology and the number of differentiated immune cells. VSGC12 also showed a higher potency than fluticasone furoate in repressing most asthma symptoms. Finally, VSGC12 showed a better side effect profile than fluticasone furoate at their respective effective doses, including better insulin response and less bone loss in an animal model. The excellent therapeutic and side effect properties of VSGC12 provide a promising perspective for developing this potent glucocorticoid as a new effective drug for asthma.

## Introduction

Asthma is a chronic inflammatory disease characterized by reversible airflow obstruction and bronchospasm [1]. Asthma affects an estimated 300 million people worldwide, with 250 000 annual deaths attributed to the disease [2]. In the US, asthma affects about 1 in 12 people (8%) and causes more than $50 billion in medical cost [3]. Inhaled glucocorticoids are the most common and effective treatment for asthma. In particular, the combination of glucocorticoids (anti-inflammatories) and β_2_-adrenergic receptor agonists (bronchial dilators) has achieved great success in preventing and controlling asthma attacks [4]. However, glucocorticoid inhalation for the treatment of asthma still encounters two major issues. First, in severe disease cases, some patients respond poorly to inhaled glucocorticoids [5]. Second, long-term use or high dose use of inhaled glucocorticoids causes many unwanted side effects, such as diabetes and bone loss, even though delivery by inhalation has fewer side effects than systemic administration [6, 7].

Glucocorticoids act through direct binding to the glucocorticoid receptor (GR), a ligand-regulated transcription factor of the nuclear receptor family. GR has two transcriptional activities, activation (transactivation) and repression (transrepression). Generally, the beneficial effects of glucocorticoids are primarily mediated by GR’s activity in transrepressing major inflammation pathways such as the NF-κB and AP-1 pathways [8]; in contrast, the unwanted side effects are mostly mediated by GR’s transactivation of anabolic (for example, gluconeogenic) and osteoclast pathways [9, 10]. However, the boundaries between transactivation/unwanted and transrepression/beneficial effects are sometimes blurred in certain circumstance. For example, GR-mediated inductions of *GILZ* and *MKP1* genes have been shown to contribute to the anti-inflammation activity of glucocorticoids [11, 12]. Clinical studies also show that many side effects of glucocorticoids are associated with a high dose use of glucocorticoids. For instance, a threshold pattern of hypertension and glaucoma was observed when prednisone was prescribed at more than 7.5 mg per day [13]. Normally, the side effects associated with high-dose glucocorticoids mainly come from two sources: one is the off-target activation of related steroid hormone receptors, such as the mineralocorticoid receptor (MR) [14]; the other is the transactivation activity of GR. Numerous observations suggest that genes induced by GR generally require a higher glucocorticoid dose than genes repressed by GR [15–17]. For example, the IC50 of fluticasone propionate (FP) to inhibit granulocyte macrophage colony-stimulating factor release is 1.8×10^−11^ M, while its EC50 for the induction of the β2-adrenergic receptor is 1.1×10^−9^ M in A549 cells [17]. These observations suggest a dosage window at which glucocorticoids can induce the beneficial transrepression activity without evoking the unwanted transactivation activity of GR.

To minimize systemic effects, glucocorticoids for asthma treatment are delivered by inhalation at a very low dose (50–100 μg) relative to that of oral glucocorticoids (5–10 mg) [6, 18]. This requires highly potent glucocorticoids that can deliver much greater local (lung bronchi) efficacy than ones with low potency, to quickly and maximally suppress the inflammation response in the lungs to prevent an asthma attack. Thus, the most successful glucocorticoids for asthma treatment are those with a high potency [4], such as FP, fluticasone furoate (FF) and mometasone furoate (MF). Particularly, FF has enhanced GR-binding affinity and has been shown to have improved efficacy in certain cases of moderate and severe asthma [19–21].

Our previous structure work on the ligand-bound GR ligand-binding domain (LBD) revealed key determinants of glucocorticoid potency [22]. A structural comparison of the cortisol-bound GR LBD with the dexamethasone (DEX)-bound GR LBD [23] revealed that a C1−C2 double bond in the steroid A ring stabilizes the hydrogen network of the 3-ketone group of the ring with key residues (R611 and Q570) of the GR ligand-binding pocket (LBP) [22]. Further, our MF-bound GR LBD structure unveiled a key mechanism to robustly boost glucocorticoid potency through complete occupancy of a hydrophobic cavity in the GR LBP by the lipophilic C-17α furoate ester group of MF [22]. Applying this structural insight, we have converted a low potency glucocorticoid into the potent compound VSG22, which has a dramatically increased potency and efficacy relative to the parental compound [22]. Further modification of VSG22 based on additional structural insight gained from the structures of the cortisol- and DEX-bound GR LBD allowed us to generate a highly potent glucocorticoid, VSGC12, which has ideal properties for asthma treatments reported in this paper.

## Results

### Structure-based design of the high affinity glucocorticoid VSGC12

**Figure 1:**
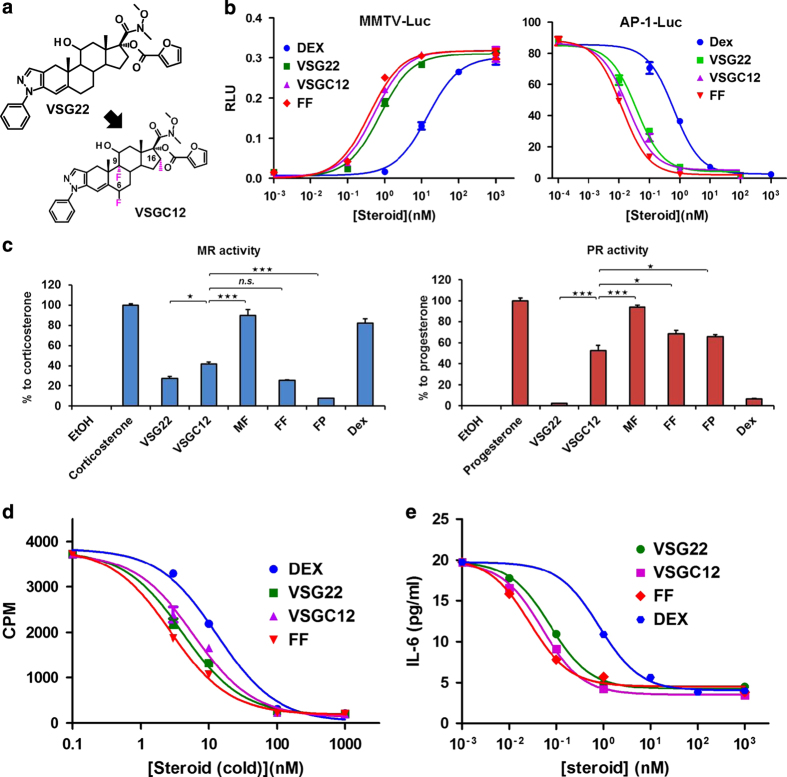
VSGC12 is a highly potent glucocorticoid. (**a**) Chemical structure of VSGC12. (**b**) VSGC12 has potency higher than VSG22, but lower than FF, in reporter assays in AD293 cells. Left panel, MMTV induction reporter assay; right panel, AP-1 repression reporter assay. RLU, relative luciferase units. (**c**) An examination of off-target effects in MR and PR reporter assays. Steroid concentration: 1 μM. Data were plotted as percent of that at 1 μM native ligands (corticosterone for MR, progesterone for PR). *P<0.05; **P<0.01; ***P<0.001, n.s., not significant; *n*=3. (**d**). An *in vitro* H^3^-Dex competition binding assay for examining ligand-binding affinity to GR. Radioactive ligand H^3^-DEX: 15 nM. The IC_50_ for DEX, VSG22, VSGC12 and FF are 13.4, 4.1, 6.1 and 2.7  nM, respectively. (**e**) A cytokine inhibition assay for testing the ability of candidate ligands to suppress cytokine IL-6 release from A549 cells. The IC_50_ for DEX, VSG22, VSGC12 and FF are 0.80, 0.07, 0.05 and 0.03 nM, respectively.

The MF-bound GR LBD structure revealed a key to improving glucocorticoid potency via introducing a furoate ester at the C-17α position of the steroid D ring. Applying this principle, we were able to create the highly potent VSG22, which has a more than 1 000-fold increase in potency relative to the original backbone VSG24. Further comparison of the DEX-bound GR LBD structure with the cortisol-bound GR LBD structure revealed that the F atom at the C-9 position and the methyl group at the C-16 position also contribute to the affinity by increasing the binding surface in the LBP of GR [22]. Incorporating these modifications, as well as an F atom at the C-6 position, to VSG22 created a novel glucocorticoid, VSGC12 (Figure 1a). We used cell-based reporter systems to examine the potency of VSGC12 for both induction (MMTV-Luc) and repression (AP-1-luc and NF-κB-Luc) in AD293 cells, a well-established cell line for glucocorticoid-mediated reporter assays [22, 24]. We first compared the activity of VSGC12 with VSG22, the backbone of VSGC12 and then compared VSGC12 with DEX, a gold standard glucocorticoid, and with FF, the most potent reported glucocorticoid [25]. In the MMTV reporter assay, VSGC12 showed an increased potency relative to VSG22, and an activity lower than, yet still close to, FF (Figure 1b, left panel). The induction EC_50_ for DEX, VSG22, VSGC12 and FF were 14.4, 0.74, 0.51 and 0.38 nM, respectively. In the AP-1 reporter assay, VSGC12 also showed higher potency than VSG22, but lower one than FF (Figure 1b, right panel). The repression IC_50_ for DEX, VSG22, VSGC12 and FF were 0.62, 0.04, 0.02 and 0.01 nM, respectively, consistent with the general observation that induction requires a higher dose than repression. Similar results were obtained using the NF-κB as reporter, which yielded IC_50_ values for DEX, VSG22, VSGC12 and FF of 1.08, 0.10, 0.08 and 0.04 nM, respectively (Supplementary Figure S1). MR and progesterone receptor (PR) are the closest orthologs of GR in the steroid hormone family and their activations by glucocorticoids at high dose are the major glucocorticoid off-target effects [14, 26]. We examined the off-target activity of VSGC12 at the saturation concentration of 1 μM, in comparison with the clinically most used inhaled glucocorticoids FP, FF and MF. The data showed that VSGC12 has a higher MR activity than VSG22 and FP, but a lower activity than MF and a similar activity (difference not statistically significant) as FF (Figure 1c, left panel). For PR activity, VSGC12 showed a higher activity than VSG22, but a lower activity than MF, FF and FP (Figure 1c, right panel). In an *in vitro* binding assay, VSGC12 has a higher binding affinity for exogenously expressed GR than DEX, but a lower affinity than FF and VSG22 (Figure 1d). A cytokine inhibition experiment in lung cancer A549 cells, a human lung epithelial cell line that has been generally used to evaluate the ability of glucocorticoids to inhibit the secretion of secondary inflammatory cytokines on primary pro-inflammatory cytokine stimulation [27], showed that VSGC12 has higher potency than VSG22, but a lower potency than FF, in inhibiting the release of IL-6 (Figure 1e), similar to what we found in the reporter assays.

### VSGC12 has a strong anti-inflammation activity in mouse macrophage cells

**Figure 2:**
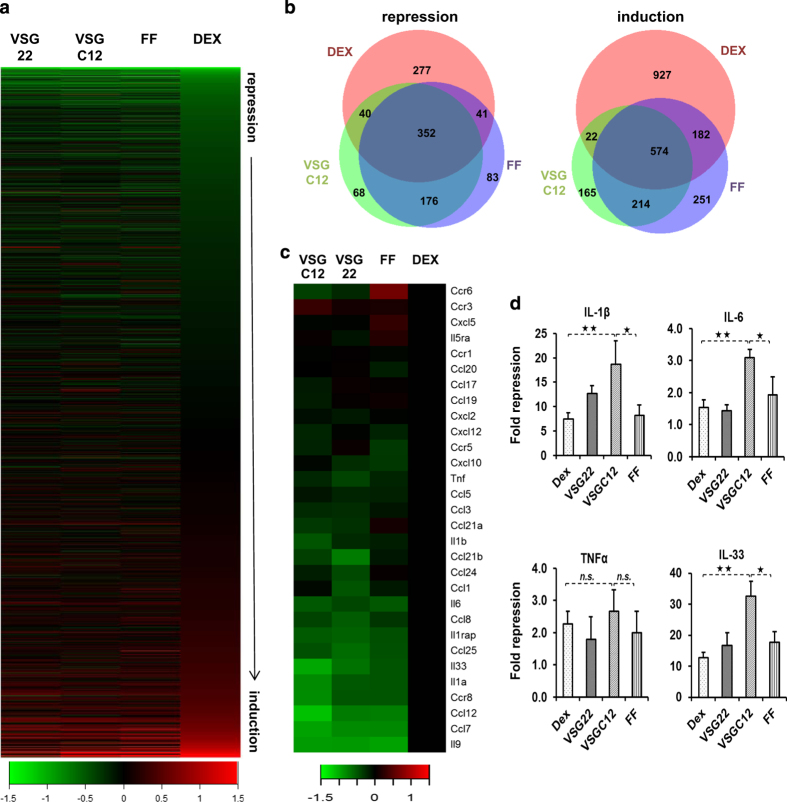
Gene expression profile of VSGC12. (**a**) A microarray gene expression analysis of VSGC12, VSG22, FF and DEX in mouse macrophage RAW264.7 cells on the induction of an inflammation response. Inflammation was induced by 1 μg ml^–1^ LPS at a steroid hormone concentration of 100 nM. Data were plotted as relative expression level to vehicle (DMSO), and aligned to the gene expression pattern seen upon DEX treatment, from most downregulated to most upregulated genes. (**b**) Venn diagrams of genes induced or repressed more than twofold in RAW264.7 cells. (**c**) Gene expression profile of pro-inflammatory cytokines in RAW264.7 cells. Data were normalized to DEX as 0. (**d**) A validation of gene expression in RAW264.7 cells by quantitative PCR. Error bars indicate s.d., *n*=3, statistics analysis is based on ΔΔCt, *P<0.05; **P<0.01, n.s., not significant.

Glucocorticoids are the most effective anti-inflammation agent because they repress almost all major inflammation signals. Macrophage cells have a key role in the initiation of the inflammation response and are also a main target of glucocorticoids for their anti-inflammation activity [28]. To examine the overall anti-inflammation activity of VSGC12, we used RAW264.7 cells, a mouse macrophage cell line, to profile the global gene expression pattern in response to VSGC12, VSG22, DEX and FF. We used LPS to elicit the inflammation and then treated with various glucocorticoids to investigate their anti-inflammation activity. Aligned with DEX, from most downregulated genes to most upregulated genes, VSGC12, VSG22 and FF followed the overall same trend as DEX, with the exception of some different signatures (Figure 2a). Venn diagrams for induction and repression show that VSGC12-, FF- and DEX-regulated genes highly overlap. Of a total of 710 genes repressed by DEX more than twofold, about half (352) were also repressed both by VSGC12 and FF (Figure 2b); similarly about half of the genes induced by DEX (574) were also induced by both VSGC12 and FF. A pathway analysis of common genes regulated by VSGC12, VSG22, FF and DEX shows that the two most repressed pathways are the MAPK signaling pathway (Kegg pathway # mmu04010) and cytokine−cytokine receptor interaction pathway (Kegg pathway # mmu04060) (Supplementary Table S1), consistent with the anti-inflammation activity of these steroid compounds. Especially, the pro-inflammatory cytokines of the cytokine−cytokine receptor interaction network are triggers of inflammation response, and their inhibition is the key for anti-inflammation treatment [29]. A close look at the inhibition activity of these pro-inflammatory cytokines, normalized to DEX (Figure 2c, black column), shows that VSGC12, VSG22 and FF have much stronger inhibition activity than DEX (Figure 2c). In particular, VSGC12 showed strong repression for the four key pro-inflammatory cytokines, IL-1β, IL-6, TNFα and IL-33. We validated these results by quantitative PCR, which showed that VSGC12 has a much higher repression activity on IL-1β, IL-6 and IL-33 than FF and DEX (Figure 2d), consistent with the microarray data, suggesting that VSGC12 may have a highly enhanced anti-inflammation activity *in vivo*.

### VSGC12 outperforms FF in a murine asthma model

FF has an enhanced GR-binding affinity compared with other clinically used inhaled glucocorticoids [25, 30]. It is considered to be the most potent glucocorticoids for asthma treatment based on its improved anti-inflammation activity in both animal models and clinical studies [19, 20, 25, 31, 32]. It outperformed FP, the best-selling asthma drug (Advair, GSK) in certain categories, such as better respiratory cell protection [31] and longer lung retention [30, 33], and in certain circumstance it showed an improved treatment effect in patients with moderate and severe disease [20, 21]. Generally, owning to its high potency, a FF dose of 100 μg once a day delivers the same treatment effect as 250–500 μg FP twice a day for the treatment of asthma [20, 32, 34].

**Figure 3:**
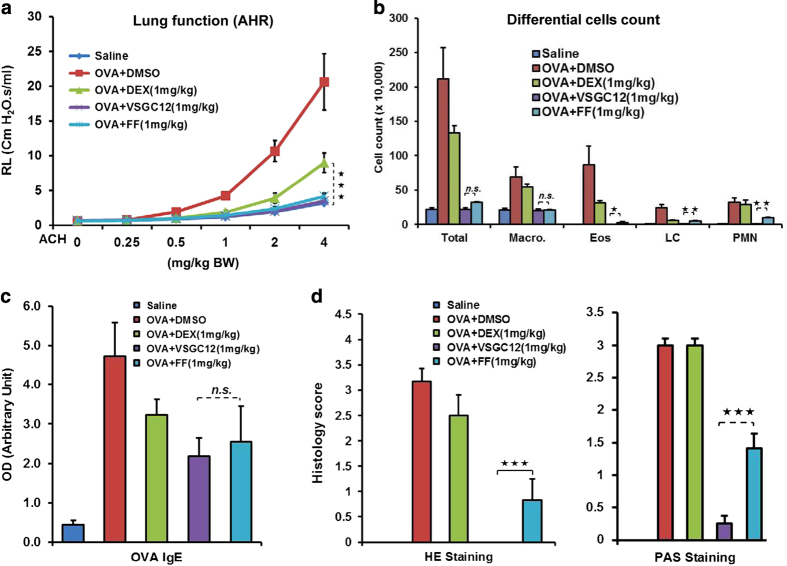
Examination of the efficacy of VSGC12 in a mouse OVA-induced asthma model. (**a**) Lung function (AHR) of BALB/c mice with different treatment. RL, resistance of lung, cm H_2_O.s/ml. ACH, acetylcholine. (**b**) Differentiated cells count. Macro, macrophage; Eos, eosinophils; LC, lymphocytes; PMN, phagocytic monocytes. (**c**) OVA-specific IgE. OD, arbitrary unit=(OD_405_–OD_540_)×dilution factor. (**d**) Histology score, left panel, hematoxylin and eosin (HE) staining; right panel, PAS staining. Error bars indicate s.e.m., each group *n*=10. *P<0.05; **P<0.01; ***P<0.001; n.s., not significant.

We first examined the efficacy of our candidate compounds (VSG22 and VSGC12) in a mouse ovalbumin (OVA)-induced acute asthma model in comparison with DEX, the standard glucocorticoid. We chose Balb/c mice, the most commonly used mice for allergic asthma model, to do the study [35]. For easy handling in rodents, we delivered the treatment by conventional intraperitoneal (i.p.) method. At a relative high dose (1 mg kg^–1^), our preliminary data showed that neither VSG22 nor DEX can effectively repress the airway hyper-responsiveness (AHR), a critical indicator of asthma in the mouse model; on the other hand, VSGC12 robustly repressed AHR to the basal level (Supplementary Figure S2). We therefore decided to only focus on VSGC12. In a further comparison to FF, the leading clinical compound for asthma treatment, both VSGC12 and FF delivered a maximal repression activity to strongly suppress the AHR to the basal level at the dose of 1 mg kg^–1^, largely surpassing DEX (Figure 3a). Since differentiated immune cells (macrophage, eosinophils, lymphocytes and phagocytic monocytes) are the indicators of lung inflammation, we examined these cells in the lungs of mice in response to different treatments. Similar to AHR, both VSGC12 and FF strongly repressed the infiltration of immune responsive cells into the lungs (total and differential cell count), while DEX had only a mild effect (Figure 3b). A close look at the treatment effects of VSGC12 and FF showed that VSGC12 actually outperformed FF in many aspects of the phenotype such as eosinophils, lymphocytes, phagocytic monocytes count (Figure 3b) and histology (Figure 3d). VSGC12 also showed better improvement indicators of lung function than FF, including total differentiated cell count and OVA-inducted serum IgE level (Figure 3c), but these differences were not statistically significant. Of particular interest is the histology score, which was reduced on VSGC12 treatment to almost zero for hematoxylin and eosin staining and to 0.25 for periodic acid–Schiff (PAS) staining. In contrast, FF treatment yielded only a moderate score (0.83 for hematoxylin and eosin stain and 1.5 for PAS stain; Figure 3d), indicating that VSGC12 has the ability to completely repress inflammation close to the basal level in the lungs.

### VSGC12 displays a highly improved potency in repressing asthma symptoms in a mouse asthma model

**Figure 4:**
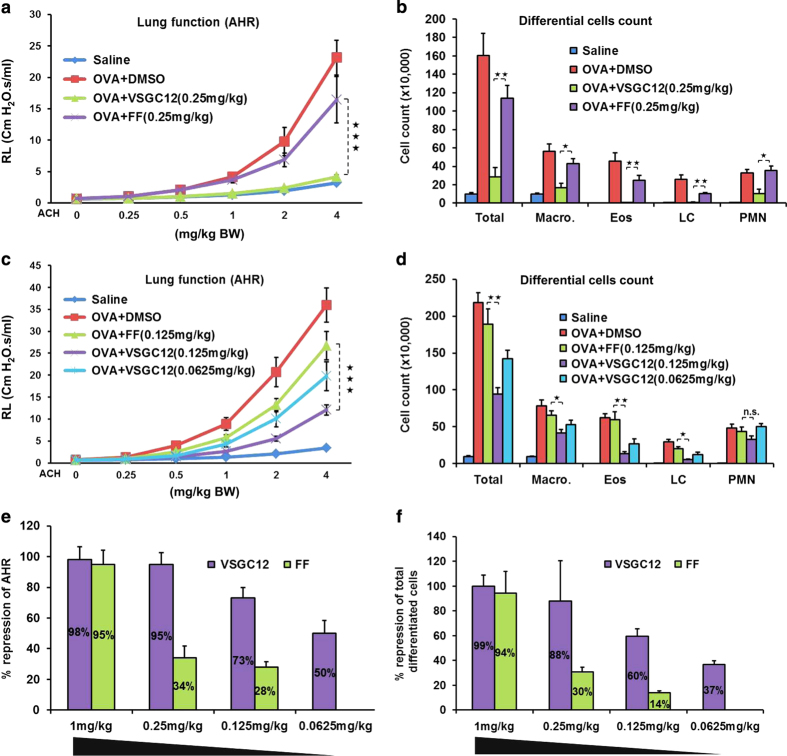
Examination of the potency of VSGC12 in a mouse OVA-induced asthma model. (**a**) Lung function of BALB/c mice treated with 0.25 mg kg^–1^ VSGC12, or FF. (**b**) Differential cells count in the lungs of mice treated with 0.25 mg kg^–1^ VSGC12 or FF. (**c**) Lung function of BALB/c mice treated with the indicated amounts of VSGC12 or FF. (**d**) Differential cell count in the lungs of mice treated with the indicated amounts of VSGC12 or FF. (**e**) A side-by-side comparison of the repression activities of various doses of VSGC12 and FF on AHR. Data were plotted as percent of repression at the maximal ACH challenge of 4 mg kg^–1^, with vehicle control (OVA+DMSO) as 0 and saline as 100. (**f**) A side-by-side comparison of the repression activity of VSGC12 and FF at various doses on total differential cell count. Data were plotted as percent of repression between saline (100) and OVA+DMSO (0). Error bars indicate s.e.m., each group *n*=8. *P<0.05; **P<0.01; ***P<0.001.

The stronger anti-inflammation activity delivered by VSGC12 at a relatively high dose indicated that VSGC12 may have a higher potency than FF in the mouse asthma model. To test this hypothesis, we decreased the dose to 0.25 mg kg^–1^. At this dose, VSGC12 still delivered a maximal repression activity (>95%) on AHR (Figure 4a), while FF lost most repression activity (<35%). Further decreasing the VSGC12 dose to 0.125 mg kg^–1^ still resulted in over 70% repression activity; and even at 0.0625 mg kg^–1^, VSGC12 retained 50% repression activity (Figure 4c and e), while FF at 0.125 mg kg^–1^ had <28% repression activity. Similar results were also seen in the lung inflammation (total differential cell count) in which 0.25 mg kg^–1^ VSGC12 delivered a maximal repression and 0.125 mg kg^–1^ VSGC12 still delivered an effective repression (Figure 4b and d). In the histology evaluation, 0.25 mg kg^–1^ VSGC12 showed a much lower score than 0.25 mg kg^–1^ FF in both hematoxylin and eosin and PAS staining (Supplementary Figure S3a). At even lower doses (0.125 or 0.0625 mg kg^–1^), the differences between VSGC12 and FF on histology were not significant (Supplementary Figure S3c). For unknown reason, the OVA-IgE levels of VSGC12 and FF at lower doses (0.25, 0.125, 0.0625 mg kg^–1^) were unexpectedly higher than the vehicle control (Supplementary Figure S3b and S3d). A replot of AHR repression of all dose of VSGC12 and FF at the maximal challenge of acetylcholine (4 mg kg^–1^), indicated that the minimal dose for VSGC12 to maximally repress the inflammation is 0.25 mg kg^–1^, and the minimal dose for VSGC12 to effectively repress AHR is 0.0625 mg kg^–1^, while the effective FF dose to maximally repress the inflammation is 1 mg kg^–1^ (Figure 4e). Similar results were also obtained by the total differentiated cells count (Figure 4f). These data indicate that VSGC12 is more effective at low dose than FF in the mouse asthma model, which makes VSGC12 highly valuable for a potential clinical investigation.

### VSGC12 has fewer side effects than FF at the effective dose for asthma treatment

The dosage difference between induction and repression (induction needs a higher concentration than repression), together with the fact that most off-target side effects (for example, MR activation) can only be induced at a high dose of glucocorticoids, suggests the possibility of developing a highly potent glucocorticoid that can be used at a low dose to effectively repress inflammation without evoking the unwanted side effects. To test this hypothesis, we measured the major side effects (growth inhibition, insulin resistance and bone loss) of VSGC12 at a low dose in mouse models. We chose DBA/1 mice to do the side effects study based on the fact that not only the glucocorticoid-induced insulin resistance can be easily developed in this strain [36], but also the glucocorticoid caused bone loss is readily to be generated in this strain [37]. Judging from the lung function AHR data, the most critical data for asthma, the minimal dose to maximally repress (maximal effective dose) lung inflammation was 0.25 mg kg^–1^ for VSGC12, and 1 mg kg^–1^ for FF (Figure 4e). We therefore mainly compared the side effects of VSGC12 with FF at the maximal effective dose (0.25 mg kg^–1^ VSGC12 vs 1 mg kg^–1^ FF). As the minimal dose for VSGC12 to effectively repress AHR (>50%) is 0.0625 mg kg^–1^, we also examined the side effect of VSGC12 at this minimal effective dose (0.0625 mg kg^–1^). In addition, we also chose 2.5 mg kg^–1^ DEX as a positive control for measuring glucocorticoid side effects based on reported data [38].

**Figure 5:**
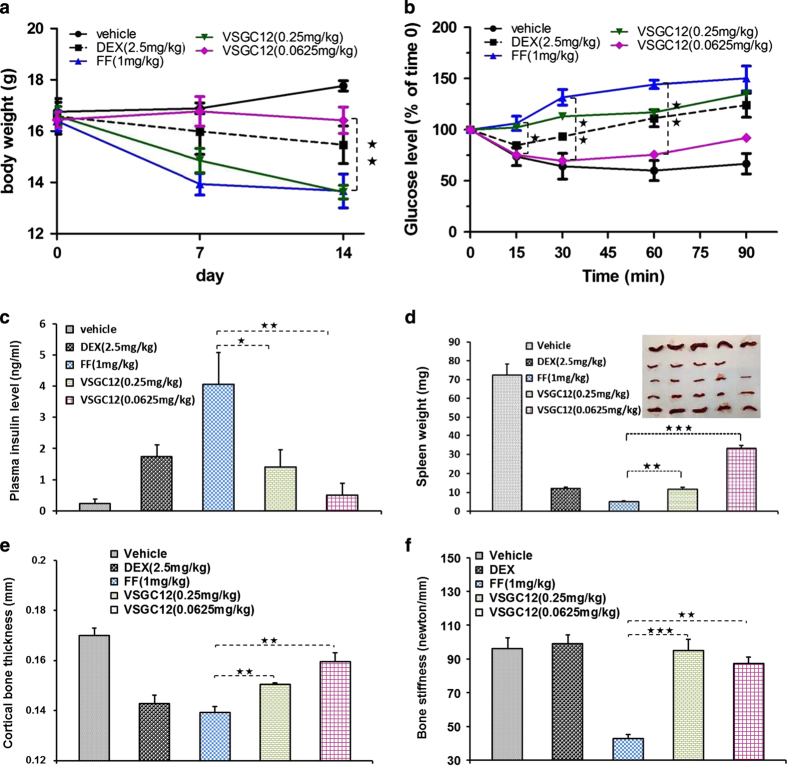
Examination of side effects of VSGC12 and FF at the effective dose. (**a**) Body weight of DBA/1 mice treated with the indicated daily dose of steroids for 2 weeks. Mice body weight was measured at day 0, 7 and 14. (**b**) Insulin-tolerance test of mice treated with the indicated daily doses of steroids for 7 days. Glucose level was measured at day 7 at various time points before and after insulin injection. Data were plotted as percent of the glucose level of time 0. (**c**) Plasma insulin levels of mice treated with the indicated daily doses of steroid for 7 days. (**d**) Spleen size and weight for mice treated with the indicated daily doses of steroid for 2 weeks. (**e**) Cortical bone thickness of femurs of mice treated with the indicated daily doses of steroid for 2 weeks. (**f**) Bone stiffness of femurs of mice treated with the indicated daily doses of steroid for 2 weeks. Mouse strain: DBA/1. Each treatment *n*=5, error bars indicate s.e.m., *P<0.05; **P<0.01; ***P<0.001.

Two weeks daily i.p. injection of 1 mg kg^–1^ of FF caused a significant growth inhibition in 7-week-old mice, suggesting that this high dose is toxic for young mice. Generally, inhalation method for asthma treatment has much less systemic exposure than i.p. injection, therefore it is unlikely that inhalation of FF will have such dramatic growth inhibition on asthma patients. However, clinical report did suggest that there is some association between a lower growth rate and a high dose use of inhaled glucocorticoids in children <17 years old [39]. VSGC12 at 0.25 mg kg^–1^ showed similar growth inhibition as FF, however, VSGC12 at its minimal effective dose of 0.0625 mg kg^–1^ did not cause a significant growth inhibition (Figure 5a). Another major side effect of glucocorticoids is diabetes, and insulin resistance is the key mechanism of glucocorticoid-induced diabetes [40]. Daily injection of 1 mg kg^–1^ FF for 7 days caused hyper-insulin resistance in an insulin-tolerance test. Although VSGC12 at 0.25 mg kg^–1^ still caused insulin resistance, the resistance was much less severe than for FF, and at 0.0625 mg kg^–1^, VSGC12 no longer induced insulin resistance (Figure 5b). Since glucocorticoid-induced insulin resistance is generally associated with an increase of endogenous insulin [36], we determined the plasma insulin level of mice treated with various doses of VSGC12 and FF. Consistent with the insulin-tolerance test data, 1 mg kg^–1^ FF caused a dramatic increase of the endogenous insulin level, while 0.25 mg kg^–1^ VSGC12 caused a moderate increase, and 0.0625 mg kg^–1^ VSGC12 did not significantly increase the endogenous insulin level (Figure 5c).

Generally, lymphoid organ atrophy is considered to be the side effect of glucocorticoid [41], however, it is also accepted that the apoptosis of lymphoid cells may contribute to anti-inflammation activity of glucocorticoid. Although the mechanism of glucocorticoid-induced apoptosis in lymphoid cells is not entirely understood, it has been suggested that the transactivation activity of GR had a role in this process [42]. We examined the effects of these compounds on atrophy of the spleen. Treatment with 1 mg kg^–1^ of FF caused a dramatic shrinkage of the spleen, while 0.25 mg kg^–1^ of VSGC12 caused a less severe atrophy and 0.0625 mg kg^–1^ of VSGC12 caused only a mild change in spleen size (Figure 5d). One of the most common side effects of glucocorticoids is bone loss. We used μCT scanning to examine the bone density of femur bones of mice treated with different steroids. The data showed that 1 mg kg^–1^ FF caused a dramatic decrease of the cortical bone thickness, while 0.25 mg kg^–1^ VSGC12 only caused a mild decrease, and 0.0625 mg kg^–1^ did not cause a significant decrease compared with vehicle treatment (Figure 5e). Consistent with these data, 1 mg kg^–1^ FF caused a dramatic decrease of bone strength in terms of stiffness, while VSGC12 at both 0.25 mg kg^–1^ and 0.0625 mg kg^–1^ no longer caused damage to bone strength (Figure 5f). Taken together, at the maximal effective dose, VSGC12 shows a better side effect profile than FF on insulin response and bone loss, and at the minimal effective dose, VSGC12 shows fewer side effects in all categories we analyzed (growth inhibition, insulin resistance, spleen atrophy and bone loss) than the gold standard DEX. This, together with its highly enhanced therapeutic effects in the mouse asthma model, suggests that VSGC12 has significant promise for its development into a better treatment for asthma patients.

## Discussion

Inhaled glucocorticoids are the cornerstone of asthma therapy. Most asthma symptoms can be well controlled in a majority of patients by inhaled glucocorticoids, or combinations of an inhaled glucocorticoid and a β_2_-adrenergic receptor agonist. However, 5–10% of asthma patients respond poorly or are basically non-responsive to the currently available glucocorticoid treatment; usually these patients have severe or very severe disease [5]. Although patients with uncontrolled asthma are a minority of the total asthmatic population, they constitute most disability and mortality of all asthma patients and use a large portion of economic resources and health-care services, including emergency visits and hospitalizations [43]. Even though treatment with protein agents (for example, antibody-based therapy) may be the answer to uncontrolled asthma in the future [44], highly potent glucocorticoids such as FF have been shown to improve the lung function of a subset of patients with uncontrolled asthma [20]. Especially, the combination of FF with vilanterol, a long-acting β2-adrenergic receptor agonist, has been shown to improve the treatment adherence in certain patients with chronic obstructive pulmonary disease [32], a much more severe certain disease than asthma, suggesting that developing a highly potent glucocorticoid may be an important approach to combat uncontrolled asthma or chronic obstructive pulmonary disease.

VSGC12 had been designed and developed based on our knowledge of the structural basis of glucocorticoid potency. Our previous structural study of glucocorticoid potency revealed three keys to improve glucocorticoid potency [22]. First, the stable hydrogen network interaction between the steroid A ring and the key residues of the LBP of the GR LBD; second, the full occupancy of the hydrophobic cavity via a lipophilic furoate ester at the C-17α position of the steroid D ring; third, the fine-tuning role of modifications at the C-6, C-9 and C-16 positions to precisely positioning the ligand in the LBP of GR. Applying these principles, we have generated the highly potent VSGC12. Although our *in vitro* binding assays with exogenously expressed GR shows that VSGC12 has lower binding affinity than FF (Figure 1d), our reporter assay and cytokine inhibition assay demonstrate that the potency of VSGC12 in cells is close to that of FF (Figure 1b and e), and, most importantly, superior in *in vivo* animal studies. In the mouse asthma model, VSGC12 outperformed the current leading glucocorticoid FF in many aspects, suggesting that VSGC12 has an enhanced potency in the lung bronchi and thus holds significant promise for the treatment of asthma patients.

Like all corticosteroid treatments in inflammatory diseases, a major concern of inhaled glucocorticoids is their unwanted side effects caused by prolonged or high dose use. These side effects hamper further use of glucocorticoids in many chronic inflammatory diseases like asthma and arthritis. The concept of dissociated glucocorticoids that deliver the beneficial anti-inflammation activity but not the unwanted side effects might be the ultimate answer to those problems in the future. Up till now, limited progress has been made in developing novel dissociated glucocorticoids that are both fully dissociated and highly potent. Also, it has been reported that certain GR-induced genes (that is, *GILZ* and *MKP1*) actually contribute the anti-inflammation activity of GR [11, 12], suggesting that even the completely dissociated glucocorticoids may still compromise certain beneficial activity of GR. Our cell-based reporter assays agree with the general observation that gene induction by GR requires a higher dose than gene repression by GR (Figure 1b and Supplementary Figure S1). These observations suggest the existence of a dosage window between transactivation and transrepression, which may provide an alternative way to minimize the side effect through the development of a highly potent glucocorticoid that delivers an effective treatment at a dose that is too low to induce many major side effects. This strategy may be particularly effective for asthma treatment as the inhalation method provides an additional buffer to minimize the exposure of glucocorticoid to major side effect organs such as liver, bone and muscles.

In supporting this hypothesis, VSGC12 delivered a maximal anti-inflammation activity at one quarter the dose of FF (0.25 mg kg^–1^ vs 1 mg kg^–1^), and showed reduced harmful effects relative to FF in certain aspects such as insulin response and bone loss. Although lack of 0.5 mg kg^–1^ dose of FF, judging from the AHR activity decay rate of VSGC12 of equal activity (0.25 mg kg^–1^, 95% repression equal 1 mg kg^–1^ FF) in Figure 4e, it is unlikely that 0.5 mg kg^–1^ FF will retain full repression activity, thereby, we feel fair to compare 0.25 mg kg^–1^ VSGC12 with 1 mg kg^–1^ FF. An interesting observation in our mouse study of glucocorticoid-induced insulin resistance is that the degree of resistance (glucose level) is nicely correlated with the increased endogenous insulin level. One general hypothesis for this is that high dose of glucocorticoid decreases the sensitivity of insulin and pushes the body to secrete more insulin to control the blood glucose level, continuous injection of high dose of glucocorticoids forces this vicious cycle and eventually leads the collapse of insulin response and becomes diabetic [40]. On further decreasing the dose, VSGC12 still retained a number of treatment effects against asthma symptoms without causing significant side effects compared with the DEX standard (Figure 4e and Figure 5). These properties make VSGC12 a promising candidate for asthma treatment.

In summary, we have developed the highly potent glucocorticoid VSGC12 for asthma treatment based on our structural study of glucocorticoid potency. VSGC12 outperformed the leading clinical compound FF in both efficacy and potency in many aspects of asthma in a mouse model and showed a reduced side effect profile compared with FF at the effective dose. These results suggest that VSGC12 is a strong drug candidate for asthma treatment.

## Materials and Methods

### Cell based reporter assay

Similar to previous reports [22, 24], for induction 100 ng pHHLuc (MMTV-Luc) plasmid, 0.1 ng pRShGR together with 5 ng phRGtkRenilla were transfected by X-tremeGENE 9 (Roche) into AD293 cells per well in 24-well plate. For transrepression, 10 ng AP-1-Luc or 10ng pNF-κB-luc, 100 ng pRShGR and 5 ng phRGtkRenilla were transfected into AD293 cells per well. We used the same transfection protocol for the MR and PR assays, except of the use of 0.1 ng MR or PR plasmid to substitute GR. One day after transfection, for induction, cells were directly treated by steroids for 16 h; for repression, cells were treated with either PMA at 0.6 ng ml^–1^ (for AP-1 reporter) or TNFα at 2 ng ml^–1^ (for NF-κB reporter), together with various steroids. Cells were collected by addition of 1× Passive Lysis Buffer (Promega, Madison, WI, USA), and luciferase activity was assayed by the Dual-Glo Luciferase system (Promega). Data were plotted as firefly luciferase activity normalized to Renilla luciferase activity in Relative Luciferase Units.

### *In vitro* GR ligand-binding assay

The *in vitro* GR binding assay is similar to the one described previously [22]. Basically, [^3^H]-Dex at 15 nM was incubated with 5% GR cytosol plus 20 mM sodium molybdate in TAPS buffer (pH 8.8) and the indicated concentrations of unlabeled competitors. Data were plotted as a standard competition curve by GraphPad Prism 5.

### Cytokine inhibition assay

A549 cells into 24-well plates were split at 40 000 cells/well. After 1 day growth, cells were treated with various steroids 1 h before overnight induction of inflammation by 2 ng ml^–1^ TNFα according to literature [27]. Sixteen hours after treatment, cell culture supernatants were subjected to anti-IL-6 Elisa (R&D Quantikine Elisa Human IL-6 Kit) according to the manual.

### Total RNA extraction and quantitative PCR

Mouse macrophage RAW264.7 cells were grown in 6-well plates to reach 80% confluence. The cells were pretreated with different steroids at 100 nM 1 h before overnight induction of inflammation by 1 μg ml^–1^ LPS. Total RNA was isolated using a Qiagen mini RNA extraction kit (Valencia, CA, USA). Complementary DNA was synthesized from total RNA with an Invitrogen Superscript cDNA synthesis kit (Carlsbad, CA, USA). Target genes were quantified with a Power SYBR Green Real-Time PCR kit (Carlsbad, CA, USA) (ABI) in a StepOnePlus real-time PCR instrument (Applied Biosystems, Carlsbad, CA, USA). In every case, GAPDH (glyceraldehyde-3-phosphate dehydrogenase) was used as an internal control and data were analyzed by the ΔΔCt method. The specificity of target primers was tested both in a dissociation (melting) curve (Supplementary Figure S4) and against a water control. Primer sequence information is included in Supplementary Figure S4.

### Microarray analysis of gene expression

RAW264.7 cells were treated with designated steroids at 100 nM 1 h before overnight LPS stimulation of inflammation. Each treatment was duplicated. The following day, RNA samples were isolated using a Qiagen mini RNA extraction kit and applied to the Agilent Whole Mouse Genome 8x60K gene expression platform. Data were analyzed by the limma package [45] in R program. Heat maps were generated by the HeatMap 2 package. Pathway analysis was done via DAVID Bioinformatics Resources 6.7 (NIAID) and Venn diagrams were generated by BioVenn [46].

### Animal studies

All animal studies were approved by the Institutional Animal Care and Use Committee of Van Andel Institute and University of California, San Francisco, and were performed in accordance with the ethical guidelines of the Animal Welfare Act.

### OVA-induce mouse asthma model

As described before [47, 48], 7–9-week-old female Balb/c mice (Taconic) were sensitized on days 0, 7 and 14 by i.p. injection of 50 μg OVA/1 mg alum in a total of 200 μl saline. Control mice received an equal volume of alum only. Mice were lightly anesthetized by isoflurane inhalation and challenged with intranasal OVA (100 μg in 30 μl saline) or saline on days 21–23, and were i.p. injected with different steroids. Twenty-four hours after the last challenge and treatment, pulmonary resistance was measured in response to a range of concentrations of intravenous acetylcholine using the forced oscillation technique with the FlexiVent system (SCIREQ) as previously described [48]. Serum samples were analyzed for OVA-specific IgE using an ELISA (BD Biosciences, clone R35-118). Total and differential cell counts were determined by hemocytometer and by light microscopic evaluation of more than 300 cells per slide as previously described [48]. After lavage, lungs were inflated with 10% buffered formalin to 25 cm H_2_O and transferred to 10% buffered formalin. Sections (5 μm) were stained with H&E for semi-quantitative assessment of inflammation and PAS for evaluation of mucus production. To quantify inflammation, H&E-stained lung sections were de-identified for blinding and scored for peribronchial and perivascular inflammatory cell infiltration: grade 0, no infiltration; grade 1, <25% of examined area; grade 2, 25–50%; grade 3, 51–75%; and grade 4, >75%. To quantify goblet cell hyperplasia, PAS stained lung sections were de-identified for blinding and scored for the percentage of PAS positive cells among airway epithelial cells: grade 0: none; grade 1 <25% of airway epithelial cells; grade 2, 25–50%; grade 3, 51–75%; and grade 4, >75%.

### Insulin-tolerance test

DBA/1 mice (Taconic) were weighted just before the experiment. After 4 h fasting, mice were i.p. injected with 0.75 U kg^–1^ of insulin (Huamlin R). Blood samples were taken from the tail at time 0 (before insulin injection), 15, 30, 45, 60, 90 and 120 min after injection. Plasma glucose levels were measured using an AlphaTrak glucose meter. All data were plotted as percent of glucose level at time 0. Endogenous insulin levels were measured using the Ultra Sensitive Mouse Insulin ELISA kit (Crystal Chem, Downers Groove, IL, USA).

### Bone density and biomechanics testing

Femur bones were collected and scanned by μCT (Skyscan 1172 uCT) for quantification of trabecular and cortical bone parameters. The thresholds for μCT quantification of trabecular and cortical bone parameters were set at 80–100 and 100–110, respectively. μCT analyses of cortical bone parameters were performed on 100-μCT slices (1 mm total) from the middle shaft of femurs; trabecular parameters were assessed in 100-μCT slices (1 mm total) immediately below the distal growth plate of the femur. Following uCT scans, the femurs were individually tested on a TestResources 570 Force Transducer. Bones were thawed in PBS before being tested, to ensure no brittleness was due to freezing. Using a four-point bending apparatus, with an upper length of 3.5 mm and lower length of 7.3 mm, the bones had a force applied to them until breaking. Data points were collected at a frequency of 50 Hz and a deflection rate of 0.005 mm s^–1^.

### Statistical analysis

All mouse data were expressed as means±s.e.m. (*n*≥5 samples). Reporter assays, binding assays and real-time PCR data were expressed as means±s.d. of triplicate samples (*n*≥3). Data were analyzed by two-tailed, unpaired *t*-tests with GraphPad Prism 5 or Excel software.

## Accession codes

The microarray data are processed for submission to the National Center for Biotechnology Information Gene Expression Omnibus database (Access number: GSE73733).
