# Supplementary material for: Discovery of a highly potent glucocorticoid for asthma treatment
Source: Cell Discov. 2015 Dec 15;1:15035–. doi: 10.1038/celldisc.2015.35 (PMC4822341; doi:10.1038/celldisc.2015.35)
Supplement: Supplementary Figure S1 [file celldisc201535-s1.pdf]

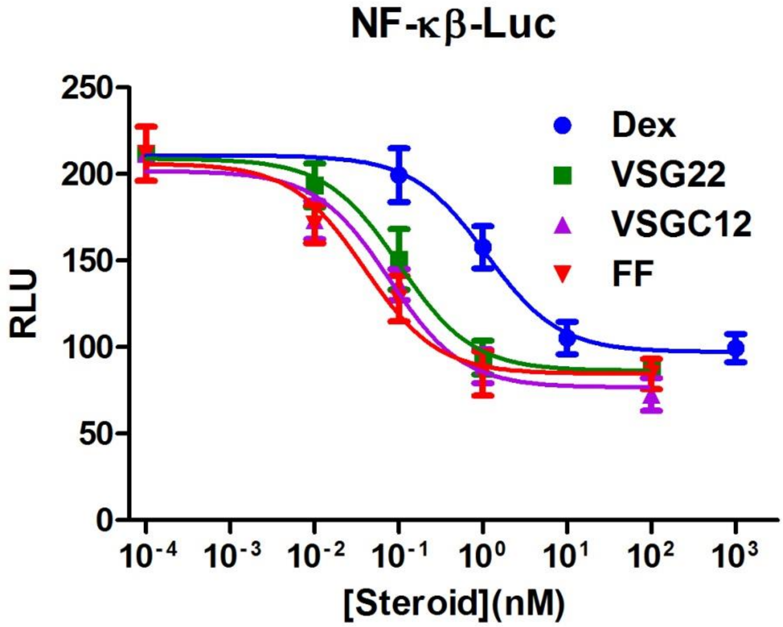

Supplementary figure 1

Supplementary Figure S1. A NF-κβ repression reporter assay to examine the repression activity of the indicated steroids. The NF-κβ activity was induced by TNFα at 2 ng/ml before adding various steroids at designated doses. Cells, AD293. Error bars indicate SD, n=3.
