# Supplementary material for: Discovery of a highly potent glucocorticoid for asthma treatment
Source: Cell Discov. 2015 Dec 15;1:15035–. doi: 10.1038/celldisc.2015.35 (PMC4822341; doi:10.1038/celldisc.2015.35)
Supplement: Supplementary Figure S2 [file celldisc201535-s2.pdf]

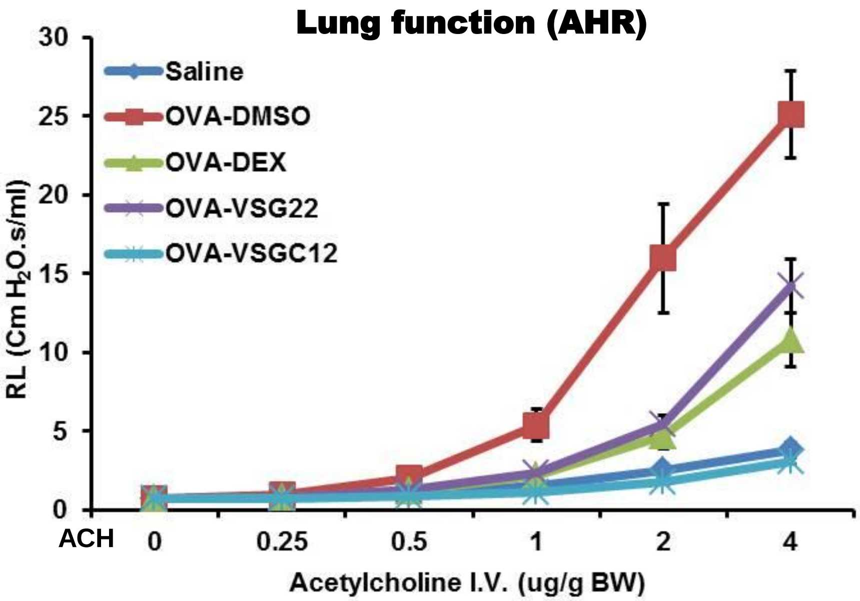

Supplementary figure 2

Supplementary Figure S2. Lung function (AHR) of BALB/c mice with designated steroids at 1 mg/kg or vehicle control. RL, resistance of lung, cm H_2O.s/ml. ACH, acetylcholine. Each treatment n=8, error bars indicate S.E.M.
