# Supplementary material for: Discovery of a highly potent glucocorticoid for asthma treatment
Source: Cell Discov. 2015 Dec 15;1:15035–. doi: 10.1038/celldisc.2015.35 (PMC4822341; doi:10.1038/celldisc.2015.35)
Supplement: Supplementary Figure S3 [file celldisc201535-s3.pdf]

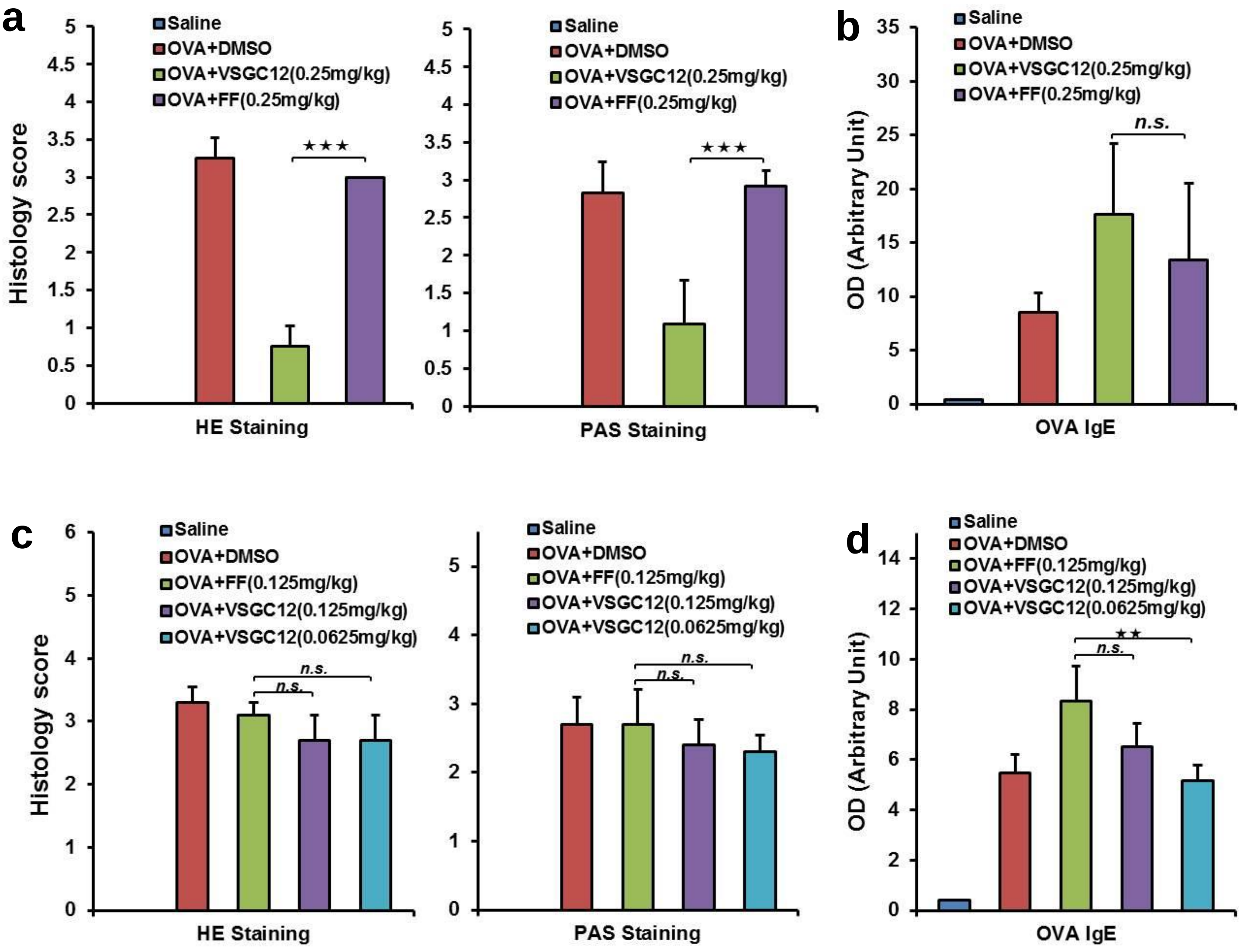

Supplementary figure 3

Supplementary Figure S3. Histology score and OVA-IgE level of BALB/c mice treated with various steroids at various dose in the OVA-induced asthma mouse model. (a) Histology score of BALB/c mice treated with 0.25 mg/kg VSGC12 or FF. Left panel, HE staining; right panel, PAS staining. (b) OVA specific IgE level of BALB/c mice treated with 0.25 mg/kg VSGC12 or FF. (c) Histology scores of BALB/c mice treated with 0.125 mg/kg VSGC12, 0.125 mg/kg FF or 0.0625 mg/kg VSGC12. Left panel, HE staining; right panel, PAS staining. (d) OVA specific IgE level of BALB/c mice treated with 0.125 mg/kg VSGC12, 0.125 mg/kg FF or 0.0625 mg/kg VSGC12. Error bar indicates S.E.M, each group n=8. ★, $p<0.05$; ★★, $p<0.01$; ★★★, $p<0.001$; n.s., not significant.
