# Supplementary material for: Discovery of a highly potent glucocorticoid for asthma treatment
Source: Cell Discov. 2015 Dec 15;1:15035–. doi: 10.1038/celldisc.2015.35 (PMC4822341; doi:10.1038/celldisc.2015.35)
Supplement: Supplementary Figure S4 [file celldisc201535-s4.pdf]

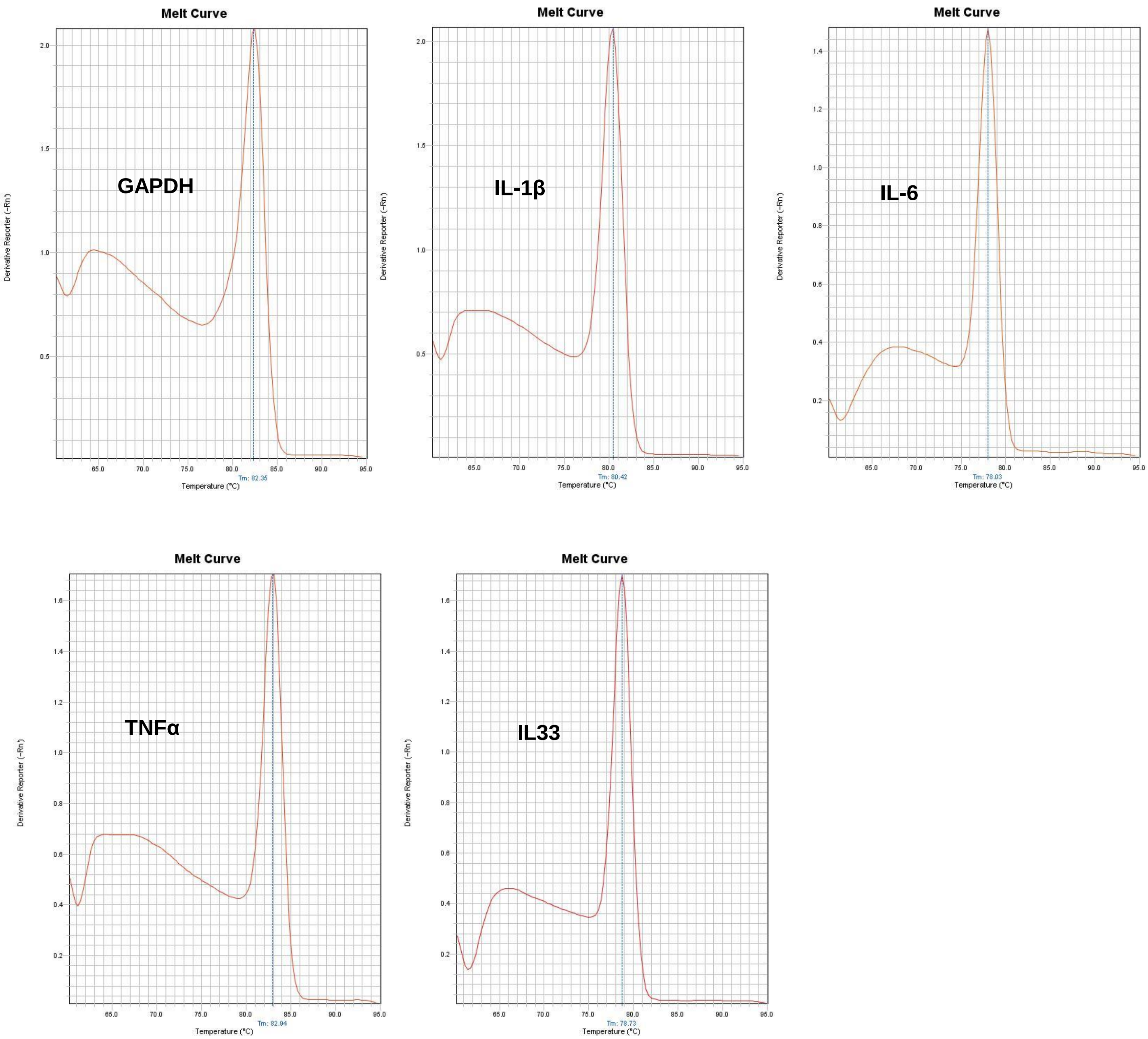

Sequence of primers for qPCR

gene	Forward primer	Reverse Primer
GAPDH	AGGCCGGTGCTGAGTATGTC	GCAGTTGGTGGTGCAGGATG
IL-1β	GCCCATCCTCTGTGACTCAT	AGGCCACAGGTATTTTGTCG
IL-6	CCGGAGAGGAGACTTCACAG	TTCTGCAAGTGCATCATCGT
TNFα	GAACTGGCAGAAGAGGCACT	AGGGTCTGGGCCATAGAACT
IL-33	GCTGCGTCTGTTGACACATT	TGATTGACTTGCAGGACAGG

Supplementary figure 4

Supplementary Figure S4. Melt curves and sequences of primers used for quantitative PCR to measure the mRNA levels of target genes in R264.7 cells. Upper panels, melt curve; lower panel, primer sequences.
