# Supplementary material for: Discovery of a highly potent glucocorticoid for asthma treatment
Source: Cell Discov. 2015 Dec 15;1:15035–. doi: 10.1038/celldisc.2015.35 (PMC4822341; doi:10.1038/celldisc.2015.35)
Supplement: Supplementary Table S1 [file celldisc201535-s5.pdf]

Supplementary Table S1. Top pathways downregulated by DEX, VSG22, VSGC12 and FF. Data were analyzed by DAVID Bioinfomatics Resource 3.7 (NIAID). Top two pathways: MAPK signaling pathway, KEGG pathway # (Kegg pathway # mmu04010) and cytokine-cytokine receptor interaction (Kegg pathway # mmu04060) pathway (for pathway detail information see http://www.kegg.jp/).

Top downregulated pathways

Term	Count	%	PValue	List Total	Pop Hits	Pop Total	Fold Enrichment	FDR
MAPK signaling pathway	20	0.561167	1.14E-05	136	265	5738	3.184239734	0.012972
Cytokine-cytokine receptor interaction	14	0.392817	0.004629	136	244	5738	2.420805207	5.123428
Ether lipid metabolism	5	0.140292	0.008718	136	35	5738	6.027310924	9.449656
Systemic lupus erythematosus	8	0.224467	0.01038	136	103	5738	3.27698458	11.15546
Pathways in cancer	15	0.420875	0.019018	136	323	5738	1.959342561	19.55984
p53 signaling pathway	6	0.16835	0.022607	136	69	5738	3.668797954	22.83367
Hypertrophic cardiomyopathy (HCM)	6	0.16835	0.047124	136	84	5738	3.013655462	42.14255
Endocytosis	10	0.280584	0.047818	136	202	5738	2.088672103	42.61795
NOD-like receptor signaling pathway	5	0.140292	0.057348	136	62	5738	3.402514231	48.80226
Dilated cardiomyopathy	6	0.16835	0.064863	136	92	5738	2.751598465	53.24297
alpha-Linolenic acid metabolism	3	0.084175	0.06576	136	18	5738	7.031862745	53.74858
Jak-STAT signaling pathway	8	0.224467	0.066347	136	152	5738	2.220588235	54.07736
Viral myocarditis	6	0.16835	0.069812	136	94	5738	2.693053817	55.97236
Glycerophospholipid metabolism	5	0.140292	0.072153	136	67	5738	3.148595259	57.21249
Bladder cancer	4	0.112233	0.07524	136	42	5738	4.018207283	58.79862
Long-term depression	5	0.140292	0.088717	136	72	5738	2.92994281	65.11508
Arrhythmogenic right ventricular cardiomyopathy (	5	0.140292	0.099462	136	75	5738	2.812745098	69.50398

Supplementary table 1
